# Intermittent Fever

**Published:** 1899-06

**Authors:** Frank W. Patch

**Affiliations:** South Framingham, Mass.


### PROCEEDINGS OF THE SOCIETY OF HOMŒOPATHICIANS,

### BUREAU OF CLINICAL MEDICINE.

F. W. PATCH, *Chairman.*

### INTERMITTENT FEVER.

FRANK W. PATCH, M. D., SOUTH FRAMINGHAM, MASS.

A list of fifteen cases treated during the season of 1897; with comments. Reported for the Society of Homœopathicians.

Case 1.—Mrs. S. J., fifty years, dark complexion, stout, nervous temperament; attack in afternoon or evening; beginning in upper extremities; shaking, restlessness; despondent, loquacious delirium, talks of killing herself; at one attack left the house and was found wandering toward a brook, where she said she would drown herself; wanted to be held while shaking; thirst, bitter vomiting, severe headache, worse from light and noise; sweat on face and hands; peculiar mapped tongue appearing like a ringworm. After Lycopodium, the chills ceased for a time, only to return in a week or so with redoubled vigor. Stramonium, CM (F.), proved curative.

Case 2.—Miss E. P. B., fifty years, stout, light complexion, of inferior mental development; tertian. Paroxysms in the afternoon, beginning with cough; creepiness and shivering without real shaking; headache with throbbing; bitter vomiting; drinking aggravates nausea; heat, with flushed face; thirst; sensitive to odors of food; desire for open air; tongue red and clean; owing to the want of intelligence in this patient, it was particularly difficult to obtain reliable symptoms; the cough and severe vomiting seemed the ruling features. Ipecac, CM (F.), was given with a palliative result. Gradual improvement went on in what seemed an orderly manner, for two weeks when the symptoms returned with daily paroxysms. Pulsatilla, CM (F.), cured.

Case 3.—A. F., unmarried woman of forty years; thin, light complexion; tertian; anticipating. Chill, bitter vomiting during or at close; mostly in hands and arms; cough; yawning; heat, with flushed face; nausea; sleepiness; sweat, with headache, vertex, and temples as though screwed in a vise; no thirst in any stage; dislike of talking; throbbing in head; aggravation of general feelings from acids. Ferr-met., CM (F.).

Case 4.—Mrs. H., seventy years, dark complexion, nervous, loquacious temperament; irregular tertian. Chill consisting of coldness without shaking, not always perceptible to nurse; thirst; tongue with broad, dry centre, like chip of wood; drinking nauseates though thirsty; burning of feet; greenish vomiting; pain in right arm; headache through temples; retching; involuntary urination; incoherent talk; rolling of head from side to side; sensation as though apart and could not get herself together again; burning in stomach; red pepper-like sediment in urine. Bell., CM (F.).

Case 5.—Miss H., age forty, dark complexion; tertian; afternoon paroxysms. Prodrome; thirst; headache in vertex and eyes; chill, with shaking above knees; restless, irritable; desire to be alone; aggravation of headache from noise and light; catching pain in left hypochondrium on deep breathing; at first thirst in chill for ice water, which aggravated, later no thirst in chill; heat, with severe headache and throbbing in vertex, as though eyes would be pushed out of head; intense, burning thirst; mental confusion; covered, sleep, sweat from below upward; thirst for hot drinks; uncovered, sweat at night in sleep; stiffness of muscles of neck and back on waking each morning; hives appearing night before chill.

The full picture of this interesting case did not come out well at first, the chill, as noted, being absent and many of the other symptoms indistinct. Nat-mur., CM (F.), served to reduce it to a beautiful state of order, the shaking chill then coming out with severity, and the way seeming to be made clear for the curative remedy, Ignatia, CM (F.).

Case 6.—C. B., three years; plump, medium complexion, sallow skin; anticipating, tertian; afternoon. Chill with cold extremities, blue fingers; quiet; thirst before and during chill; heat and sweat with thirst and restlessness; lachrymation; perspiration of head; fretful. Nat-mur., CM (F.).

Case 7.—Mrs. E. O. D.; seventy years. This case reported two years ago as an unsatisfactory one, making a doubtful recovery on Nux-vom. This year returned as was expected sooner or later. It should not have been reported before. Chill in forenoon, anticipating, tertian, preceded by pain in back and legs; thirst; tongue with broad, white centre; wandering delirium throughout intense heat; no sweat. Hyos., CM (F.).

Case 8.—M. C.; about seventy years; a weak-minded old man, said to have been disappointed in love in young days; hardly competent to answer questions intelligibly; tertian; forenoon; only shivering chill; frontal headache; nausea; thirst; flushed face in heat; trembling; twitching of muscles about mouth; stupid, half-sleeping condition, perfectly quiet, from which only a grunt could be elicited in rousing. Phos-ac., CM (F.).

Case 9.—H. S.; child of sixteen months; light complexion; tertian, afternoon. Chill with shaking; goose-flesh; sleepiness; nails purple; paleness; quiet; no thirst; heat, with flushed face; gasping respiration; palpitation visible; sweat profuse on head, neck, and face; impatient; dark circles under eyes; skin yellowish. Arsenicum, CM (F.).

Case 10.—F. S.; active, wiry man of thirty-five; dark complexion; tertian, anticipating. Chill very severe, with thirst; covered, face purple, ringing in ears; tongue white; heat and sweat following in order with thirst. Nat-mur., CM (F.).

Case 11.—Mrs. G. T.; forty-five years; full habit, reddish complexion; tertian, anticipating attacks. Chill, beginning in arms, going up and down back; nausea, bitter vomiting; thirst, vomits soon after drinking; catching pain in left hypochondrium and in epigastrium through to back, worse from jar, breathing, etc.; thirst for large quantities of ice water in heat; vertigo on rising; tongue, broad white centre; red, dry edges, dry mouth; backache; faintness; intense burning heat, with great physical restlessness and mental agitation. Arsenicum, CM (F.).

Case 12.—Mrs. J. B. McC.; fifty years; stout, plethoric, dark complexion; tertian, changing to quotidian. Chill with shaking; covered; cough; aching of left shoulder, preceded by left-sided headache; vomiting of bile; heat, with mild delirium; flushed face; chilly, if uncovered; much thirst; restlessness; sweat, profuse, sticky; hydroa about mouth, tongue dry, brown centre, white edges.

This was one of the most trying cases imaginable, and reflects little credit on the prescriber. Search after search was made for the simillimum, with no further result than palliation or some slight modification of symptoms until, on the indication of the time of chill, Gamboge, CM (F.), was given and the attacks ceased for a week or more. It seemed hardly possible that this could be permanent and, sure enough, it was not; everything returned as before. This time another and more careful examination revealed the fact that for six months previous to this attack the patient had suffered from a watery diarrhœa, worse during the day, with sleepiness in the middle of the day, before dinner. Aloes, CM (F.), made a prompt and lasting cure. It seems strange that, even after years of experience with this disease, one will neglect to get the totality of symptoms at the start. These lessons must be learned over and over again by the hard knocks of failure. Often the full picture does not develop at the beginning of a case of intermittent for the need of an antipsoric remedy or, through natural sluggishness of the system, but there is no reason why old symptoms should not be ferreted out, except when one is so blinded by what is immediately before him that more important things are lost sight of.

Case 13.—Illustrates another feature in the treatment of these cases, the need of paying strict attention to the psoric features of the case in hand in order that the acute remedy indicated may have opportunity to properly do its work. Mrs. D. N.; forty-five years; dark complexion, thin, wiry, irritable, nervous temperament; tertian, anticipating. Chill beginning in hands; numbness of hands; coldness of extremities; backache; pain in legs; nausea, sour vomiting; heat, with thirst for large quantities; restlessness; vertigo; sweat, only when sleeping after the attack; tongue with broad, white centre, red tip; hydroa about mouth; tongue trembles on protrusion; restless sleep after 2 A. M.; impatient and irritable. Nux-vom. was given with great confidence here in the early part of the case, waited for, and yet no result. Then other studies were made, and several other remedies used without avail. Finally a dose of Sulphur was prescribed, which apparently of itself offered no check to the progress of the chills, but Nux-vom., CM (F.), was then given again with a most satisfactory and prompt cure following. Here the antipsoric should have been given in the first place, and would have been had the nature of the case been thoroughly understood. The next case further illustrates the same principle.

Case 14.—A. S.; forty-eight years; slight build, dark complexion, easy-dispositioned man. First attack, July 15th, chill, with vertigo; foul tasting mouth; great thirst; flushed face; intense burning heat after slight chill; dry, tickling cough, hoarseness; dull backache. Bryonia, CM (F.), promptly relieved, and after a few days the patient was out and went away for a vacation. On the day of his return, before getting into the house, he was taken with a most severe chill, and it was at once seen that a tedious fight was to be expected. The Bryonia, of course, failed to do anything now, except to make one undecided whether to let it alone or work out the case anew. The picture finally presented was as follows: Chill, with shaking all over; cough before and during sensation of coldness for some time before shaking begins; vomiting; heat, with thirst; backache; nausea after drinking, and when lying on right side; face flushed; faintness, amounting nearly to collapse at times; throbbing head; sweat, with chilliness and cold feet, must be covered; no thirst; tongue with heavy, yellow coat; face, sallow, brownish; belching after eating. Sepia, CM (F.).

Case 15.—A. H.; young woman of twenty; tertian, anticipating type; prodrome; thirst; nausea; paroxysm begins with nausea and backache; hands purple in chill; intense, throbbing headache in vertex and nape in heat, worse from motion; impatient; face flushed; drinking aggravates nausea; feet very cold; pillow seems hard; sleep immediately follows heat; tongue white, red tip; continual aching of the neck, wakes every morning with stiffness of the muscles of the nape, and aching from nape to spine; belching immediately after eating.

This was another trying case where a better knowledge of materia medica would probably have saved much discouragement for the physician and many suffering days for the patient. It seemed at first as though Nat-mur. would surely make quick work of it, but, to my surprise, it seemed to do nothing, not even a palliation followed its administration. Several other remedies were given with a similar result, when a more careful study of the symptoms seemed to show that the peculiar conditions present were the singular headache and the terrible throbbing. Glonoine was about the only remedy that just covered these symptoms, though it did not seem possible that it could cure this case. It was given and the chills ceased for a few days, together with the anticipation and the headache. It was only temporary, however, and within a week the attacks, with slight anticipation, began to creep back again, but without the head symptoms or the throbbing. Now Nat-mur., CM (F.), was given again with a beautiful cure resulting. This seems to be a singular instance of the occasional necessity of a short acting medicine, which should prepare the way for the deeper antipsoric remedy that was previously ineffective.

### DISCUSSION.

Dr. Kennedy—This is one of the papers I am always interested in. I want to ask the doctor if he has noticed the time of day of the return of the paroxysms, and if he considers that of value when he is selecting his remedy? I notice also that he does not mention thirst as being peculiar to any one of the three stages. I have had but little experience with the disease. I have been able to cure the few cases I have had.

Dr. Pease—I notice in the reports of the cases that most of them were elderly people. Is that correct, doctor?

Dr. Patch—There were two young persons. One was twenty.

Dr. Pease—Was this their first experience with the disease?

Dr Patch—I do not recollect at this moment whether any of these were second attacks or not, but I think they were all first cases.

Dr. Morgan—I do not feel that I can say anything instructive to Dr. Patch, for the cases and his management of them show such thorough investigation and careful research in finding the right remedies, that I do not think any man could improve upon them. I think he has done wonderfully good work. I have found the time of day the chill occurs of a great deal of importance. I have also found that an excellent time to give the remedy is just between the chill and the fever. Remedies act better when given then than at any other time.

Dr. Adams—I merely wish to say that I think Dr. Patch is to be congratulated on the care he has taken to adhere strictly to Hahnemann's rules. The paper is an inspiration to me.

Dr. Pease—Have you ever had a Quinine case?

Dr. Patch—I rarely find a Quinine case. In an experience of almost ten years with the disease I have seen one or two cases that have been cured by China.

Dr. Pease—Dr. Patch, have you noticed any of the conditions of locality in regard to the type? I believe I mentioned a few remedies that seemed to be most called for in certain localities. Have you investigated that?

Dr. Patch—I had three Bryonia cases a few years ago in one locality, but it may have been mere coincidence. My cases probably differ from those of the West. The disease here is apt to attack people who are not in good general health and bring out the psoric condition of the individual.

Dr. Morgan—Down near the lake in Baltimore there is a district where nearly every case I get requires Eupatorium. There is a swamp region there, and the Eupatorium grows in abundance.

Dr. Davis—I have read that in every locality would be found the remedies necessary to cure the diseases peculiar to that region. The question arose in my mind whether the Eupatorium was not the father of the disease. It is a curious thing that these plants should be where the disease predominates.

Dr. Patch—In closing the discussion, I will say that I think Dr. Kennedy will find in reading the cases that the time of day is not important in these particular instances. The majority of my cases are of what is termed the "anticipating" type, in which the time of day cannot be depended upon. I do not take it into account.

